# Investigating Leptin Gene Variants and Methylation Status in Relation to Breastfeeding and Preventing Obesity

**DOI:** 10.3390/children11111293

**Published:** 2024-10-25

**Authors:** Ayse Kilic, Sacide Pehlivan, Muhammet Ali Varkal, Fatima Ceren Tuncel, Ibrahim Kandemir, Mustafa Ozcetin, Sükran Poyrazoglu, Asli Derya Kardelen, Irem Ozdemir, Ismail Yildiz

**Affiliations:** 1 Division of General Pediatrics, Department of Pediatrics, Istanbul Faculty of Medicine, Istanbul University, 34093 Istanbul, Türkiye; ayse.kilic@istanbul.edu.tr (A.K.); mavarkal@istanbul.edu.tr (M.A.V.); ismail.yildiz@istanbul.edu.tr (I.Y.); 2 Department of Medical Biology, Istanbul Faculty of Medicine, Istanbul University, 34093 Istanbul, Türkiye; sacide.pehlivan@istanbul.edu.tr (S.P.); fatimaceren.tuncel@ogr.iu.edu.tr (F.C.T.); 3 Department of Pediatrics, Faculty of Medicine, Istanbul Health and Technology University, 34445 Istanbul, Türkiye; 4 Institute of Child Health, Istanbul University, 34093 Istanbul, Türkiye; mustafa.ozcetin@istanbul.edu.tr; 5 Division of Pediatric Endocrinology, Department of Pediatrics, Istanbul Faculty of Medicine, Istanbul University, 34093 Istanbul, Türkiye; sukran.poyrazoglu@istanbul.edu.tr (S.P.); asli.kardelen@istanbul.edu.tr (A.D.K.); 6 Istanbul Faculty of Medicine, Istanbul University, 34093 Istanbul, Türkiye; iremozdemir321@hotmail.com

**Keywords:** breast milk, leptin, epigenetics, obesity

## Abstract

**Objective:** We investigated whether the results of leptin gene (LEP) 2548G/A (rs7799039) and leptin receptor gene (LEPR) 668 A/G (rs1137101) variants, as well as the methylation analysis of CpG regions at nucleotides −31 and −51 of the LEP gene, showed any differences between breastfed and non-breastfed children in this study. **Materials and Methods:** The cross-sectional study included 100 children aged 2–5 years who were attending nursery and kindergarten and had been accepted to the Department of General Paediatrics. Infants who were exclusively breastfed for the first six months after birth constituted the study group, and those who were not only breastfeed constituted the control group. Methylation percentages at CpG islands of the LEP gene were compared between exclusively breastfed and non-exclusively breastfed infants, and the statistical significance was analyzed by looking for changes in LEP −31 and −51 nt methylation and LEP 2548G/A ve LEPR 668 A/G variants. **Results:** Both groups were compared by feeding, and the association of LEPR and LEP gene polymorphisms and −51 nt and −31 nt methylations were analyzed. There were no significant differences between the groups regarding genotype and allele frequency for the LEPR 668 A/G, LEP 2548 G/A gene variant, −31 nt methylation, and −51 nt methylation status. Similarly, there was no significant difference in genotype and allele frequency for the LEPR 668 A/G gene variant in terms of duration of exclusive breastfeeding, total breastfeeding, body mass index, family obesity, and satiety status. However, maternal support from family elders and physical activity increased the 51 nt methylation, but this methylation was not significantly affected by BMI, age, or satiety status. **Conclusions:** Maternal support from family elders and physical activity were associated with increased 51 nt methylation, but this methylation was not significantly affected by BMI, age, or satiety status. However, there are not enough studies in this area to reach a definitive conclusion, and further research is needed.

## 1. Introduction

Breast milk is an unrivaled food that meets the needs of babies until the age of six months. Breastfeeding provides optimal nutrition during the immediate postnatal period and later in life compared to formula feeding and is a reliable safeguard against obesity [1,2,3]. Metabolism and obesity are affected by genetic and environmental factors. Although the mechanisms of metabolic programming are not well defined, there is increasing evidence that epigenetic mechanisms can modulate the metabolic programming of a baby by adjusting gene expression and affecting the phenotype later in life [4,5,6]. These epigenetic effects can change cell- and tissue-specific gene expression, and this change can be transmitted from generation to generation. Epigenetic research in the pediatrics field can contribute to revealing epigenetic consequences on developmental programming, developing epigenetic biomarkers for the early diagnosis of diseases, identifying pediatric individuals at risk of disease in adulthood, and developing preventive or curative measures.

In Turkey, although the rate of mothers breastfeeding their babies immediately after birth is 90%, this rate drops to 10% at the end of the 6th month. However, evidence-based scientific studies show that exclusive breastfeeding protects babies from obesity if continued for up to 6 months. However, the genetic and epigenetic grounds have begun to be addressed more in recent years [7,8,9,10,11,12,13,14]. Although there are few studies on this subject in the literature, there are almost none in Turkey.

Leptin is a protein hormone product of the leptin (LEP) gene, identified by Zhang et al. [15] in 1994. It is mainly produced by adipose tissue and secreted into the circulation at levels proportional to the size of fat stores. Circulating leptin activates neuropeptide circuits through its specific receptor in the brain and has an appetite-reducing effect by inhibiting the orexigenic region [16]. In addition, leptin directly affects adipose tissue by stimulating lipid catabolism and inhibiting lipogenesis [17]. The evidence gathered in recent years indicates that leptin might be a potential programming factor during the lactation period.

Although studies on the leptin hormone in Turkey are carried out in the fields of endocrine and genetics, there is no study explaining the epigenetic mechanisms of the relationship between breast milk and leptin. However, international studies have reported associations between nutrition during early infancy and epigenetic features [18], as well as the preventive effect of breastfeeding against obesity [19].

This study aims to show the relationship between the LEP gene and the leptin hormone and explain the epigenetic mechanisms of its protective effect against obesity.

## 2. Material and Methods

### 2.1. Establishment of the Study Group

We planned the research as a cross-sectional study by including 100 participants (50 for the study group and 50 for the control group, between 2 and 5 years old) from children attending Istanbul Medical Faculty Sevda Sabanci Nursery and Kindergarten as well as children who came to the General Pediatrics Department and obtained informed consent. We asked the parents about the duration of exclusive breastfeeding and whether the first feeding after delivery was with initial breast milk (colostrum) or formula. We investigated the presence of obesity and diabetes in the family (parents) and took a detailed nutritional history. Children who differed only in terms of breastfeeding duration but had no chronic disease and were not taking medication were included in the groups. We planned to measure the weight, height, body mass index, blood pressure, and waist circumference of these children and their parents.

The inclusion criteria were:

- Birth at term and with appropriate weight;
- Exclusive breastfeeding for six months (the study group);
- Feeding with formula with or without breast milk supplements (for the control group).

The exclusion criteria were:

- Presence of diseases that cause obesity like Bardet–Biedl syndrome, Cushing disease etc. (the child or the family);
- History of premature delivery;
- Presence of any chronic disease.

### 2.2. Laboratory Analyses

#### DNA Isolation from Peripheral Blood Samples

We drew 5 cc peripheral blood specimens from 100 children after the survey and preserved them in EDTA tubes. First, leukocyte isolation was performed on the blood collected from the individuals included in the study, and genomic DNA was isolated from the obtained leukocytes using a Quick-DNA Miniprep Plus Kit (Zymo Research, Irvine, CA, USA) in accordance with the manufacturer’s instructions.

### 2.3. Genotyping

In this study, genotyping of LEP −2548G/A (rs7799039) and LEPR 668 A/G (rs1137101) variants was performed using the PCR-RFLP method. HhaI restriction enzyme was used for the leptin gene (LEP) −2548G/A polymorphism, and MspI enzyme was used for the leptin receptor gene (LEPR) 668 A/G polymorphism [20,21]. Samples processed in agarose gel electrophoresis were viewed under UV light and genotyping analyses were performed.

#### Bisulfite Modification and Methylation-Specific PCR

In the first stage, bisulfite conversion was performed on the DNA samples to be analyzed for the MS-PCR method using a commercial kit and according to the manufacturer’s instructions (EZ-DNA Methylation-Gold™ Kit Zymo Research).

For the LEP gene promoter region, two pairs of primers, forward and reverse, were used for each region, belonging to the −51 nt and −31 nt CpG islands [22]. Commercially obtained unmethylated and methylated DNA samples were also used as control DNA. Bisulfite-converted DNA samples amplified using PCR were loaded onto agarose gel and viewed under UV light, and the results were recorded as methylated for samples with bands and unmethylated for samples without bands.

### 2.4. Statistical Analysis

Methylation percentages in the CpG islands of the LEP gene were compared between exclusively breastfed children and those who were not breastfed, and statistical significance was investigated by looking at LEP DNA methylation and changes in common polymorphisms in the LEP gene and LEPR gene.

We used the Mann–Whitney U test to compare two groups containing non-normal-distributed continuous data and the chi-square test to compare two groups with categorical data. Also, we used the Kruskal–Wallis test to compare three groups containing non-normal distributed data. Normal distribution was tested using the Kolmogorov–Smirnov test.

We used multivariate analysis (a binomial regression test) to assess the utmost influential variables by subjecting the factors to calculations (where the collinearity assumption was met). We also used Bayesian Kendall’s tau test to test the H0 (independence) and H1 (association) hypotheses by setting the stretched beta prior width at 1. The Jamovi 2.3.18 statistical package program was used, with the alpha error rate set at *p* < 0.05.

## 3. Results

We recruited one hundred and three children; however, three subjects were excluded as they did not give blood samples.

### 3.1. Nutrition for the First Six Months

We selected 100 children (46 breastfed and 54 mixed fed (breast milk + formula milk)), compared them by feeding style, and examined whether LEPR and LEP gene polymorphisms and 51 nt and 31 nt methylations were related.

We did not detect any statistically significant differences regarding genotype and allele frequency for the LEPR 668 A/G gene (*p* = 0.776) between the two groups (Table 1).

No significant difference was detected regarding genotype and allele frequency for the LEP 2548 G/A gene when both groups were compared (*p* = 0.160) (Table 1).

No significant difference was detected regarding 31 nt methylation status when both groups were compared (*p* = 0.248) (Table 1).

No significant difference was detected regarding 51 nt methylation status when both groups were compared (*p* = 0.828) (Table 1).

### 3.2. Duration of Exclusive Breastfeeding (DEB)

The duration of exclusive breastfeeding in the 100 selected subjects was noted, and we examined whether LEPR and LEP gene polymorphisms and 51 nt and 31 nt methylation were associated with DEB.

When looking at the duration of exclusive breastfeeding, no significant difference was found in terms of breastfeeding duration and LEPR 668 A/G gene genotypes (*p* = 0.805) (Table 2).

No significant difference was detected regarding duration and LEP 2548 G/A gene genotypes when both groups were compared (*p* = 0.510) (Table 2).

No significant difference was detected regarding duration and 31 nt methylation status when both groups were compared (*p* = 0.485) (Table 2).

No significant difference was detected regarding duration and 51 nt methylation status when both groups were compared (*p* = 0.791) (Table 2).

### 3.3. Total Breastfeeding Time (TBT)

The total duration of breastfeeding in 100 selected subjects was noted, and we examined whether LEPR and LEP gene polymorphisms and 51 nt and 31 nt methylation were associated with TBT.

Considering TBT, no significant difference was found in terms of duration and LEPR 668 A/G gene genotypes (*p* = 0.774) (Table 3).

No significant difference was detected regarding duration and LEP 2548 G/A gene genotypes when both groups were compared (*p* = 0.826) (Table 3).

No significant difference was detected regarding duration and 31 nt methylation status when both groups were compared (*p* = 0.401) (Table 3).

No significant difference was detected regarding duration and 51 nt methylation status when both groups were compared (*p* = 0.572) (Table 3).

### 3.4. Body Mass Index (BMI)

Of the 98 selected children (2 obese children were excluded from this test), 42 subjects with normal BMIs and 56 subjects with underweight BMIs were compared with each other in terms of body mass index, and we examined whether LEPR and LEP gene polymorphisms and 51 nt and 31 nt methylation were related.

No significant difference was detected regarding BMI and genotype and allele frequency for the LEPR 668 A/G gene when both groups were compared (*p* = 0.075) (Table 4).

No significant difference was detected regarding BMI and genotype and allele frequency for the LEP 2548 G/A gene when both groups were compared (*p* = 0.348) (Table 4).

No significant difference was detected regarding BMI and genotype 31 nt methylation status when both groups were compared (*p* = 0.804) (Table 4).

No significant difference was detected regarding BMI and genotype 51 nt methylation status when both groups were compared (*p* = 0.432) (Table 4).

### 3.5. Family History of Obesity

Three groups—no obesity (33 cases), obesity (26 cases), and overweight (41 cases)—were compared according to the family history of the 100 selected children, and we examined whether LEPR and LEP gene polymorphisms and 51 nt and 31 nt methylation were related.

When all three groups were compared with each other, no significant difference was detected in terms of genotype and allele frequency for the LEPR 668 A/G gene (*p* = 0.882) (Table 5).

No significant difference was detected in terms of genotype and allele frequency for the LEP 2548 G/A gene between the three groups (*p* = 0.535) (Table 5).

No significant difference was detected in terms of genotype and allele frequency for the 31 nt methylation status between the three groups (*p* = 0.640) (Table 5).

No significant difference was detected in terms of genotype and allele frequency for the 51 nt methylation status between the three groups (*p* = 0.244) (Table 5).

### 3.6. Satiation Status (Does She/He Leave the Table Feeling Full or Unsatisfied?)

Of the 100 selected children, 36 reported no, and 64 reported yes to the satiation condition. Subjects were compared in terms of these two conditions and we examined whether LEPR and LEP gene polymorphisms and 51 nt and 31 nt methylation were related.

When both groups were compared with each other, no significant difference was detected in terms of genotype and allele frequency for the LEPR 668 A/G gene (*p* = 0.585) (Table 6).

No significant difference was detected in terms of genotype and allele frequency for the LEP 2548 G/A gene between the two groups (*p* = 0.267) (Table 6).

No significant difference was detected in terms of genotype and allele frequency for the 31 nt methylation status between the two groups (*p* = 0.209) (Table 6).

No significant difference was detected in terms of genotype and allele frequency for the 51 nt methylation status between the two groups (*p* = 0.438) (Table 6).

### 3.7. Maternal Support and Methylations

We could not find a statistically significant effect of maternal support (physical and emotional) from family elders on 31 nt methylation [Bayesian Kendall’s tau: −0.142, BF10:1.13 (anecdotal evidence)], but there was very strong evidence for maternal support effecting 51 nt methylation (Bayesian Kendall’s tau: −0.258, BF10:166).

**Figure 1:**
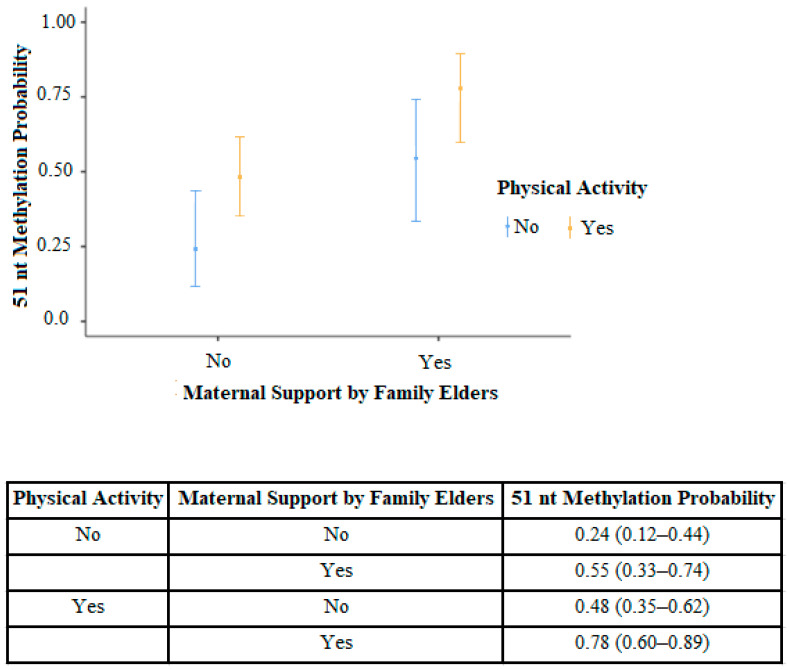
The effect of maternal support by family elders and physical activity on 51 nt methylation.

We subjected 51 nt methylations to multivariate analysis (binomial logistic regression where the collinearity assumption was met) with maternal support from family elders, BMI-SDS of the child, maternal age, child’s age, duration of exclusive breastfeeding, total breastfeeding duration, obesity history in the family, physical activity (at least two hours in the playground or two hours of active sports per day), and satiety status. We eliminated insignificant factors using the *backward elimination method*. Maternal support (*p* = 0.003) and physical activity (*p* = 0.021) remained significant confounders for the 51 nt methylation status. The graphic and estimated marginal means are presented in Figure 1. Maternal support from elder family members increased 51 nt methylation odds by 3.78 times (1.50–9.57), and physical activity increased the odds by 2.92 times (1.14–7.47).

## 4. Discussion

The benefits of breast milk for both mother and baby are a widely accepted fact [2,7,8,10,11]. Breast milk can form a link between maternal and child health [3,6,7,12]. The mother–breastmilk–baby triad is an interconnected system in which the mother’s nutrition and lifestyle affect the baby’s health [12]. Although the underlying mechanisms have not yet been fully elucidated, this relationship might be partially explained by epigenetics.

This study is the first study in Turkey addressing the positive effects of breastfeeding on obesity and it aims to explain the epigenetic mechanisms of the relationship between breast milk and the leptin gene. To our knowledge, no study in the literature examines the genetic and epigenetic relationship between breast milk, obesity, and leptin in children aged 2–5 years. We examined whether exclusive breastfeeding for the first six months, total breastfeeding time, body mass index, and family history of obesity increase the risk of obesity in preschool children, but no significant relationship was established.

Systematic studies conducted by the World Health Organization have shown the relationship between breastfeeding and obesity. This meta-analysis, which included the results of one hundred and five studies, showed that long-term breastfeeding reduced the rates of overweight and obesity [23]. Comparative studies regarding breastfeeding and its duration found that individuals exclusively breastfed and breastfed for a long time had lower obesity rates [3]. The protective effect of 7-month breastfeeding against obesity is also emphasized [24]. Other studies and meta-analyses report that breastfeeding is an important protective factor against obesity in children [2,3,25,26]. Although breast milk might be associated with epigenetic changes, these mechanisms remain unclear [9,12], and even some studies concluded that breastfeeding was not associated with obesity [27,28,29].

A new study investigating the epigenetic effects of breast milk stated that the transfer of miR-26a (a substance in breast milk) through milk might modulate the development of the baby’s adipose tissue and affect the tendency of the baby to obesity, thus raising the possibility that miR-26a might be an epigenetic regulator. This study also mentioned that the amount of miR-26a in breast milk is affected by the mother’s diet [30]. However, in the study we conducted, we did not find a significant difference regarding obesity risk in babies who were exclusively breastfed for 5 months and those who were not. The results of another meta-analysis conducted in 2005 did not show a relationship between breastfeeding and body mass index (BMI), similarly to our results [31]. In a randomized and double-blinded study conducted by Inostroza et al., breastfed babies of obese mothers grew faster than babies born to lean mothers, especially in the first six months of life [32]. In our study, obesity history in the family did not affect LEP gene methylation or polymorphisms.

A study hypothesized that breast milk contributes to the programming of the neuroendocrine system by regulating the methylation proteins of the leptin (LEP) gene promoters [33]. Leptin is a hormone that provides satiety. It regulates food intake and metabolic pathways by communicating with neuropeptides [34]. When LEP methylation decreases, leptin hormone secretion increases. Breast milk protects against childhood obesity by reducing LEP methylation and increasing leptin hormone levels [33]. Obermann et al. [35] reported that environmental and structural factors affected the epigenetic variations of the LEP gene in 17-month-old children. This methylation difference in the LEP gene was related to breast milk’s effect on obesity; however, they stated that more studies are needed in future to determine whether it can be considered a protective effect. In the study, obesity and LEP hypermethylation were proportional, but they detected low LEP methylation in children with high body mass index scores and speculated that this situation might be related to high birth weight but without a definitive conclusion. They also reported a limitation as they only included 17-month-old children and did not follow up with children of older ages when healthy weight gain could be more obvious. They also observed that LEP methylation was inversely proportional to the duration of breastfeeding, and serum leptin concentrations were significantly higher in children who were breastfed for 1–3 months [35]. Thus, a study reported that increased methylation decreased future metabolic syndrome risks by lowering serum cholesterol levels and fasting blood sugar [36]. Another study reported that methylation decreases gene transcription, and maternal high carbohydrates result in lower methylation in infants [37]. Another study stated that the connections found may depend not only on breast milk but also on the diet of the mother and the child during this period [38]. There was no significant difference between the groups regarding LEP gene methylation in our study. Neither breastfeeding duration nor exclusive breastfeeding in the first six months affected leptin gene methylation significantly. However, contrary to this finding, there are also studies showing that common polymorphisms in the LEP gene and the leptin receptor (LEPR) gene play an important role in obesity and obesity-related metabolic biomarkers [38,39,40,41,42]. However, 51 nt methylation was affected by maternal support from family elders and physical activity, independently of each other. A study from the literature on the family effect on methylations reported that maternal caregiving to children was associated with the methylations of four described genes (ZBTB22, TAPBP, ZBTB12, and DOCK4) in children [43]. Maternal diet, lifestyle, and physical activity might affect epigenetics in the antenatal period [44], but another aspect of this result was that maternal support from family elders also affected 51 nt methylation, even more than physical activity. Regarding this topic, a publication reported that maternal behaviors affected the gene expression and phenotypic differentiation of offspring by DNA methylation; thus, this transmission might affect future generations [45]. Also, a study reported that physical activity affected DNA methylations [46]. According to our results, maternal support from family elders had a positive impact on DNA methylations, far more than the effect of physical activity, and could be attributed to lifestyle or their supportive genealogical influence. Therefore, we could not provide a decisive conclusion, and we need more prospective multicenter studies to analyze the effect of maternal support from family elders.

The combined obesity and overweight rate was 2% in our study group, which is significantly lower than the 10.8% obesity rate reported in China (binomial test, *p* = 0.002) [47], the 13.8% rate in Ethiopia (binomial test, *p* < 0.001) [48] and the 26.5% rate reported in Canada (binomial test, *p* < 0.001) [49]. We attribute this situation to the low rate of exclusively formula-fed infants in our study. We believe this result alone highlights the positive effect of breastmilk on obesity.

Limitations of the study: The number of exclusively formula-fed infants is very low in our study as most of the participants had breastfeeding history, which limited the size of the control (exclusively formula fed) group. We did not measure belly circumferences and z-scores. The strength of this study is that it is one of very few studies about this topic in Turkey. Other limitations include the lack of control group for family elders and the fact that we did no evaluate leptin sensitivity.

## 5. Conclusions

As a result, it is increasingly recognized that leptin, a component of breast milk, plays a role in the postnatal programming of a healthy phenotype in adulthood. Besides its primary function in controlling body weight, leptin controls fat accumulation and body composition. Breast milk may be an essential nutrient required during lactation to ensure that the LEP gene and leptin are well organized from the early stages of development. Maternal support from family elders and physical activity increased 51 nt methylation, and methylation was not significantly affected by BMI, age, or satiety status. However, studies in this field are still not sufficient to reach a definitive conclusion; thus, comprehensive research is needed that includes the measurement of leptin, sensitivity, maternal support status, and a non-breastfed control group.

## Figures and Tables

**Table 1:** Comparison of gene variants and methylation status regarding nutrition type in the first 6 months.

		Exclusive Breastfed (*n* = 46) (%)	Breastfed + Formula (*n* = 54) (%)	*p*
LEPR 668 A/G Genotype	AA	24 (52.2%)	32 (59.3%)	0.776
	AG	19 (41.3%)	19 (35.2%)	
	GG	3 (6.5%)	3 (5.6%)	
LEPR 668 A/G Allele	A	67 (72.8%)	83 (76.9%)	0.512
	G	25 (27.2%)	25 (23.1%)	
LEP 2548 G/A Genotype	GG	12 (26.1%)	24 (44.4%)	0.160
	GA	22 (47.8%)	20 (37.0%)	
	AA	12 (26.1%)	10 (18.5%)	
LEP 2548 G/A Allele	G	46 (50.0%)	68 (63.0%)	0.065
	A	46 (50.0%)	40 (37.0%)	
LEP-Methylation 31 nt	Methylated	35 (76.1%)	46 (85.2%)	0.248
	Unmethylated	11 (23.9%)	8 (14.8%)	
LEP-Methylation 51 nt	Methylated	24 (52.2%)	27 (50.0%)	0.828
	Unmethylated	22 (47.8%)	27 (50.0%)	

**Table 2:** Comparison of gene variants and methylation status according to duration of exclusive breastfeeding.

		Duration of Exclusive Breastfeeding (*n* = 100) (%)	*p*
LEPR 668 A/G Genotype	AA	6.0 Months (3.0–6.0)	0.805 *
	AG	6.0 Months (3.6–6.0)	
	GG	5.5 Months (3.5–6.0)	
LEP 2548 G/A Genotype	GG	5.5 Months (3.0–6.0)	0.510 *
	GA	6.0 Months (3.0–6.0)	
	AA	6.0 Months (3.3–6.0)	
LEP-Methylation 31 nt	Methylated	6.0 Months (3.0–6.0)	0.485 **
	Unmethylated	6.0 Months (3.5–6.0)	
LEP-Methylation 51 nt	Methylated	6.0 Months (3.0–6.0)	0.791 **
	Unmethylated	6.0 Months (3.0–6.0)	

* Kruskal–Wallis test, ** Mann–Whitney U test.

**Table 3:** Comparison of genes variant and methylation status according to duration of total breastfeeding.

		Duration of Total Breastfeeding *n* = 100 (%)	*p*
LEPR 668 A/G Genotype	AA	9.5 Months (5.8–24.0)	0.774 *
	AG	10.5 Months (5.0–22.5)	
	GG	6.0 Months (6.0–15.0)	
LEP 2548 G/A Genotype	GG	7.0 Months (5.8–24.0)	0.826 *
	GA	11.0 Months (4.3–20.8)	
	AA	11.0 Months (6.0–24.0)	
LEP-Methylation 31 nt	Methylated	8.0 Months (5.0–24.0)	0.401 **
	Unmethylated	12.0 Months (6.0–21.5)	
LEP-Methylation 51 nt	Methylated	12.0 Months (5.0–24.0)	0.572 **
	Unmethylated	9.0 Months (6.0–23.0)	

* Kruskal–Wallis test, ** Mann–Whitney U test.

**Table 4:** Comparison of gene variants and methylation according to body mass index (BMI).

		Weakness BMI *n* = 41 (%)	Normal BMI *n* = 57 (%)	*p*
LEPR 668 A/G Genotype	AA	19 (46.3%)	35 (61.4%)	0.075
	AG	21 (51.2%)	17 (29.8%)	
	GG	1 (2.4%)	5 (8.8%)	
LEPR 668 A/G Allele	A	59 (72.0%)	87 (76.3%)	0.489
	G	23 (28.0%)	27 (23.7%)	
LEP 2548 G/A Genotype	GG	20 (35.1%)	14 (34.1%)	0.348
	GA	27 (47.4%)	15 (36.6%)	
	AA	10 (17.5%)	12 (29.3%)	
LEP 2548 G/A Allele	G	67 (58.8%)	43 (52.4%)	0.378
	A	47 (41.2%)	39 (47.6%)	
LEP-Methylation 31 nt	Methylated	47 (82.5%)	33 (80.5%)	0.804
	Unmethylated	10 (17.5%)	8 (19.5%)	
LEP-Methylation 51 nt	Methylated	31 (54.4%)	19 (45.6%)	0.432
	Unmethylated	26 (46.3%)	22 (53.7%)	

**Table 5:** Comparison of gene variants and methylation status according to family history of obesity.

		None *n* = 33 (%)	Positive *n* = 26 (%)	Overweight *n* = 41 (%)	*p* Value
LEPR 668 A/G Genotype	AA	19 (57.6%)	14 (53.8%)	23 (56.1%)	0.882
	AG	11 (33.3%)	11 (42.3%)	16 (39.0%)	
	GG	3 (9.1%)	1 (3.8%)	2 (4.9%)	
LEPR 668 A/G Allele	A	49 (74.2%)	39 (75.0%)	62 (75.6%)	0.982
	G	17 (25.8%)	13 (25.0%)	20 (24.4%)	
LEP 2548 G/A Genotype	GG	8 (24.2%)	11 (42.3%)	17 (41.5%)	0.535
	GA	17 (51.5%)	10 (38.5%)	15 (36.6%)	
	AA	8 (24.2%)	5 (19.2%)	9 (22.0%)	
LEP 2548 G/A Allele	G	33 (50.0%)	32 (61.5%)	49 (59.8%)	0.366
	A	33 (50.0%)	20 (38.5%)	33 (40.2%)	
LEP-Methylation 31 nt	Methylated	26 (78.8%)	20 (76.9%)	35 (85.4%)	0.640
	Unmethylated	7 (21.2%)	6 (23.1%)	6 (14.6%)	
LEP-Methylation 51 nt	Methylated	15 (45.5%)	11 (42.3%)	25 (61.0%)	0.244
	Unmethylated	18 (54.5%)	15 (57.7%)	16 (39.0%)	

**Table 6:** Comparison of gene variants and methylation status according to satiety status.

		None *n* = 35 (%)	Positive *n* = 65 (%)	*p*
LEPR 668 A/G Genotype	AA	22 (62.9%)	34 (52.3%)	0.585
	AG	11 (31.4%)	27 (41.5%)	
	GG	2 (5.7%)	4 (6.2%)	
LEPR 668 A/G Allele	A	55 (78.6%)	95 (73.1%)	0.392
	G	15 (21.4%)	35 (26.9%)	
LEP 2548 G/A Genotype	GG	12 (34.3%)	24 (36.9%)	0.267
	GA	18 (51.4%)	24 (36.9%)	
	AA	5 (14.3%)	17 (26.2%)	
LEP 2548 G/A Allele	G	42 (60.0%)	72 (55.4%)	0.529
	A	28 (40.0%)	58 (44.6%)	
LEP-Methylation 31 nt	Methylated	26 (74.3%)	55 (84.6%)	0.209
	Unmethylated	9 (25.7%)	10 (15.4%)	
LEP-Methylation 51 nt	Methylated	16 (45.7%)	35 (53.8%)	0.438
	Unmethylated	19 (54.3%)	30 (46.2%)	

## Data Availability

The data presented in this study are available on request from the corresponding author as we do not have permission from ethical board to publish them publicly.

## Abbreviations

LEP	Leptin gene
LEPR	Leptin receptor gene
DEB	Duration of exclusive breastfeeding
TBT	Total breastfeeding time
BMI	Body mass index
